# Impact of cyfluthrin (Solfac EW050) impregnated bed nets on malaria transmission in the city of Mbandjock : lessons for the nationwide distribution of long-lasting insecticidal nets (LLINs) in Cameroon

**DOI:** 10.1186/1756-3305-6-10

**Published:** 2013-01-11

**Authors:** Christophe Antonio-Nkondjio, Maurice Demanou, Josiane Etang, Bernard Bouchite

**Affiliations:** 1 Laboratoire de Recherche sur le Paludisme, Organisation de Coordination pour la lutte Contre les Endémies en Afrique Centrale (OCEAC), P.O. Box 288, Yaoundé, Cameroon; 2 Vector group, Liverpool School of Tropical Medicine, Pembroke Place, Liverpool, L3 5QA, UK; 3 Faculty of Health Sciences University of Bamenda, P.O. Box 39, Bambili, Cameroon; 4 Laboratoire des Arbovirus, Service de Virologie Centre Pasteur Cameroun BP 1274 Yaoundé Cameroun, membre du Réseau International des Instituts Pasteur, Yàounde, Cameroon; 5 Faculty of Medicine and Pharmaceutical Sciences, University of Douala, P.O. Box 2701, Douala, Cameroon; 6 Institut de Recherche pour le Développement (IRD), UR 016, 911, avenue Agropolis, P.O. Box 64501, 34394, Montpellier cedex 5, France

**Keywords:** Cyfluthrin, Impregnated nets, Malaria, Transmission, *Anopheles*, Cameroon

## Abstract

**Background:**

Insecticide treated materials remain the mainstay for malaria prevention. The current study reports on the entomological impact of cyfluthrin impregnated bed nets on malaria transmission in Mbandjock, a semi urban locality in southern Cameroon. Several findings pertaining to the recent distribution of LLINs across Cameroon are discussed.

**Methods:**

Malaria transmission and vector bionomics were monitored before and after impregnated net coverage. Bed nets were distributed in Mbandjock, whereas the locality of Nkoteng was free of bed nets during the entire study period. January to June 1997 represented the period before bed net coverage and September 1997 to September 1998 was the period after bed net coverage. Adult mosquitoes were collected by human landing catches. Mosquito genus and species were identified with morphological and molecular diagnostic tools. Anopheline salivary glands and ovaries were dissected to determine female infectious status and parity rates respectively.

**Results:**

A total of 6959 anophelines corresponding to 6029 in Mbandjock and 930 in Nkoteng were collected in the course of the study. Seven species were recorded in both cities : *Anopheles coustani, An. funestus, An. gambiae sl, An. moucheti, An. ziemanni, An. nili* and *An. paludis*. *An. gambiae* s.l. (>95% *An. gambiae* S molecular form) was the most abundant species representing 75.6% and 86.6% of the total anophelines caught in Mbandjock before and after bed net coverage respectively. The human biting rate (HBR) in Mbandjock decreased from 17 bites/human/night before bed net coverage to less than 4 bites/human/night during the first 7 months following impregnated bed net coverage. A significant decrease of mosquito parity rate was recorded when comparing the period before (52%) and after (46.5%) bed net distribution. The average infection rate of malaria vectors significantly decreased from 5.3% before to 1.8% after bed net coverage (p < 0.0001). The entomological inoculation rate in Mbandjock was reduced by 74% varying from 124.1 infected bites/human/year before bed net distribution, to 32.5 infected bites/human/year after bed net coverage. All entomological indexes were relatively stable in Nkoteng and no reduction of malaria transmission was recorded in this locality.

**Conclusion:**

The study confirms the effectiveness of cyfluthrin impregnated nets in reducing malaria transmission. Lessons from this study could be essential to draw guidelines for the management of the recent nationwide distribution of LLINs across Cameroon in 2011.

## Background

Over 90% of the population of Cameroon is at risk of malaria [1]. Recent reports from the World Health Organization indicate that malaria in Cameroon is responsible annually for, 30% of morbidity cases, 36% of outpatient consultation, 67% of childhood mortality and 48% of hospital admissions [2]. The economic bulk of the disease is estimated to be between 160 and 208 million USA Dollars annually [3]. Although malaria is endemic across the country, the disease is more prevalent in the equatorial forest region where transmission is perennial and attributed to: *An. gambiae*, *An. funestus*, *An. nili* and *An. moucheti*. In the sahelian and dry savanna areas, transmission is seasonal and due to *An. arabiensis*, *An. gambiae* and *An. funestus*[4]. Measures taken to combat malaria in Cameroon consist of the utilization of bed nets for prevention and the diagnosis and treatment of clinical cases [5]. The introduction of bed nets for malaria prevention started in the 1990s. Before then, vector control activities were more private initiatives. The most important large scale vector control intervention conducted in the country remains undoubtedly the indoor spraying campaigns in the 1950s. These campaigns which were conducted in pilot areas, resulted in a decrease of malaria transmission in some parts of the treated zone [6,7]. The inaccessibility of some areas particularly during the rainy season, the spread of vector resistance to insecticides and the inability of the government to provide sufficient means to maintain regular aspersions, impeded the successful implementation of the program and resulted in it being stopped in the 1960s [8]. The malaria control strategy was then reoriented on the promotion of primary health care services. The later expansion of *Plasmodium falciparum* resistant strains to chloroquine, which was used as first line treatment against malaria [9], highlighted the need to revamp vector control activities. The success of pyrethroid treated nets in the fight against malaria vectors in West Africa [10], favours its introduction as the main method of prevention from malaria attacks [8]. The initial period before bed net promotion, was preceded by small scale studies aimed at evaluating the efficacy of impregnated bed nets on vector populations and malaria transmission [8,11]. The majority of these studies demonstrated the efficacy of pyrethroid impregnated bed nets in different epidemiological settings [11,12]. Despite a growing interest for impregnated bed nets, they were still unaffordable for the majority of Cameroonians [13,14]. From 2003 onwards, several initiatives were undertaken by the Cameroonian government to increase bed net ownership. These included the free distribution of over 2 million impregnated bed nets to pregnant women and children under 5 years old, and subsidizing the cost of ITNs (insecticide-treated nets) for the rest of the population [15]. It is now estimated that about 69% of households in the cities of Douala and Yaoundé, own at least one bed net [16]. In its continuous efforts to meet its objective of significantly reducing the burden of malaria in the country, the Cameroonian government with the financial support of the Global Fund, opted for free distribution of over 8 million long lasting insecticidal nets (LLINs) to the entire population [15]. Although this initiative is largely welcomed and meets the recommendations of the World Health Organisation [17], there is a need to foresee the possible limits of this initiative in regard to previous large scale studies conducted across the country. Between 1997 and 1998 a pilot study evaluating the impact of cyfluthrin impregnated bed nets on malaria transmission, was conducted in Mbandjock a semi urban locality with over 16,000 inhabitants. About 4,000 impregnated bed nets were distributed to the population and malaria transmission was monitored before and after bed net distribution to the population. This study presents the main results recorded and, discusses the possible lessons in relation to the recent nationwide distribution of LLINs to the population in Cameroon. This study also has the objective of presenting control activities that have taken place in the country and methods used in the past. Data from past control activities in Cameroon have been desperately lacking because the majority of these studies are not published.

## Methods

### Study sites

The study took place in four districts of the city of Mbandjock (4° 19’N et 11° 54’E) namely: Gare, Membrat, Bilingue and Plateau. Mbandjock is situated 110 km north east of Yaoundé the capital city of Cameroon. Mosquito collection was also conducted in the locality of Nkoteng (4° 30’N et 12° 3’E) used as control situated some 20 km north of Mbandjock. The semi urban localities of Mbandjock and Nkoteng are situated within the Congo-Guinean phytogeographic zone characterized by a typical equatorial climate with two rainy seasons extending from March to June and from September to November. Both cities are agro-industrial towns characterized by vast zones used for sugar cane plantations. The districts of Bilingue and Membrat, are situated in Mbandjock centre and characterized by high population densities, whereas the districts of Plateau and Gare are situated at the periphery. Mbandjock is irrigated by a few rivers with the most important one being the Sanaga river passing at about 4 km away from the urban centre and, about 6 km from the centre of Nkoteng. The annual rainfall in Mbandjock and Nkoteng was estimated to be about 1500 mm.

### Adult mosquito collections

**Figure 1:**
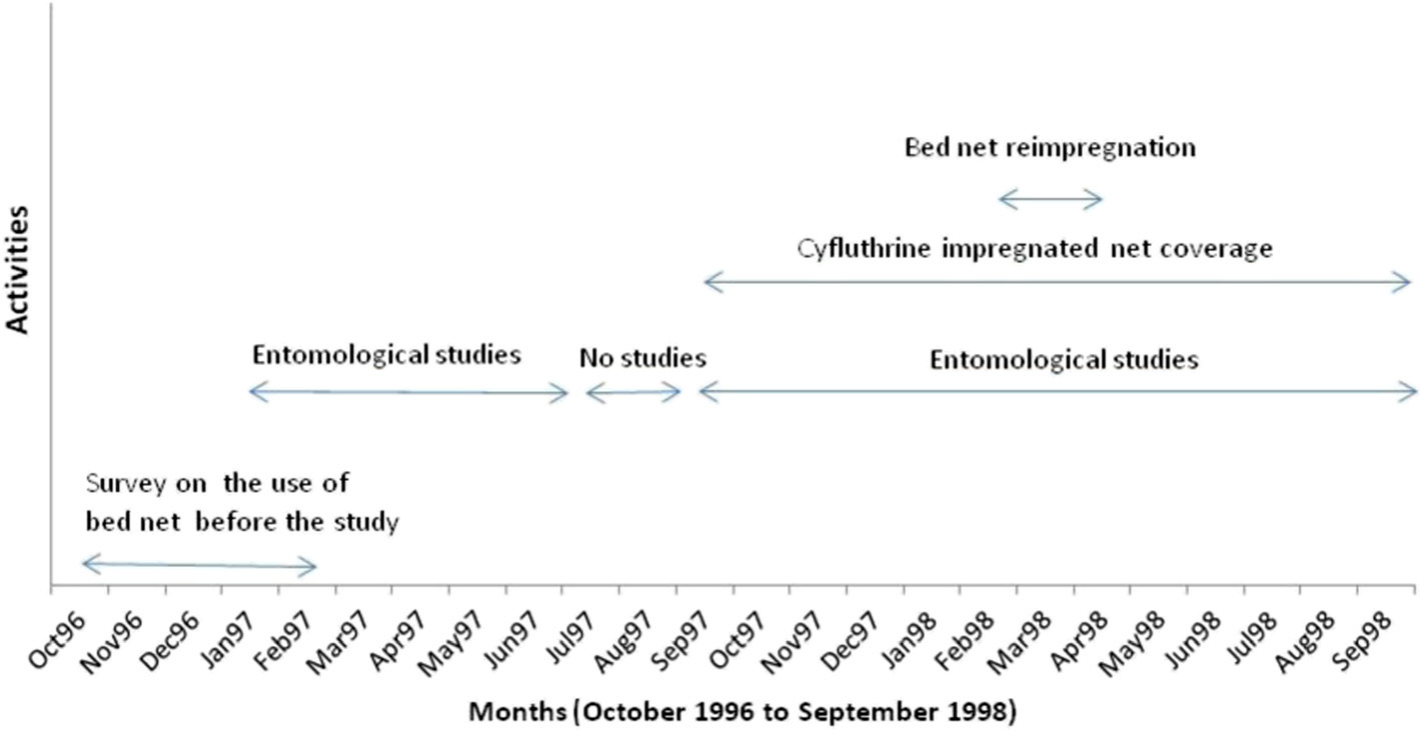
A graph showing the schedule of activities conducted during the cyfluthrin bed net coverage programme in Mbandjock.

Adult mosquitoes were collected from January 1997 to September 1998 using human landing catches from 06:00 PM to 06:00 AM. January to June 1997 represented the period before bed net coverage and September 1997 to September 1998 the period after bed net coverage (Figure 1). Mosquito collections were conducted in five sites in Nkoteng, Bilingue, Plateau and Gare and ten sites in Membrat. Selected sites ranged from 50 m for the closest to 500 m for the most distant. Collections were conducted once each month in each district. In each collection site, mosquitoes were collected both indoors and outdoors. All volunteers gave their consent for capturing mosquitoes and were given free malaria prophylaxis.

The study received the approval of the Cameroon Ministry of Health.

### Processing of mosquitoes

Anophelines were identified to species using morphological characteristics according to the identification keys of Gillies & Coetzee [18] and Gillies & De Meillon [19]. Species belonging to the *An. gambiae* complex, *An. funestus, An. nili* and *An. moucheti* groups, were further identified using available molecular diagnostic tools [20-23]. DNA extracted from a mosquito leg and/or wing was used for analysis. To assess changes in mosquito survival rate, mosquito ovaries were dissected and their parity rate determined. The infectious status of female specimens was assessed by direct observation of *Plasmodium* sporozoites in the salivary glands. About 20% of the mosquitoes detected as positive, were kept for ELISA analysis for *Plasmodium* species identification. The heads and thoraxes of some female anophelines which were detected as positive after dissection, were tested for the presence of the *Plasmodium* circumsporozoite protein (CSP) using enzyme-linked immunosorbent assay (ELISA) [24] to identify *Plasmodium* species. The sporozoroite rate was calculated as a ratio of mosquitoes infected over mosquitoes analyzed. The entomological inoculation rate (EIR) was calculated by multiplying the human biting rate estimated from landing catches by the sporozoroite rate. The parity rate was calculated as a ratio of the number of parous females over the total number of parous and nulliparous females.

### Bed net impregnation and distribution

Bed nets were distributed in the locality of Mbandjock, while Nkoteng was free of nets during the entire study period. The bed nets distributed were made of polyester fibers and originated from Thailand, Asia. Two bed net sizes were used, 16.3 m^2^ and 13.3 m^2^. They were impregnated with the target dosage of 50 mg active ingredient (a.i)/m^2^ of cyfluthrin (Solfac EW050). Bed nets were impregnated by dipping into a solution of cyfluthrin diluted with water and prepared for the impregnation of ten nets at a time. Chemical analysis of the treated nets revealed an uptake of active ingredient on nets ranging from 30.8 to 75. 1 mg a.i/m^2^ at the first treatment and 131 to 225 mg a.i/m^2^ after retreatment [25].

Impregnated bed nets were distributed to families starting from September 1997. Before bed net distribution, all families residing in the study sites were registered. People wanting to acquire bed nets were asked to pay 4 or 6$ USA per net depending on the size (the payments were spread out over a period of 4 to 8 months). The retreatment of nets was free of charge and was conducted during the months of March and April 1998.

## Results

### Net Coverage

Preliminary investigations conducted to estimate bed net coverage in study sites in Mbandjock, recorded out of a total of 1699 households, 31 bed nets used before the programme. This represents a ratio of 0.02 net per household. During the programme, a total of 2454 nets were distributed in the study sites and, 1520 for the rest of the city, making a total of 3974 bed nets distributed in Mbandjock. The proportion of net coverage varied from 65% for Membrat to 100% for Bilingue (Table 1). The low coverage rate in the district of Membrat inhabited by very poor families could be associated to the cost of the bed net, which probably did not incite families to look for means of protection. The average retreatment rate of nets was 63.8% (Table 1). No bed net in use was found in Nkoteng before the programme.

**Table 1:** Bed net utilization in Mbandjock before and during the programme

**Districts**	**N° Households**	**Pre-existing nets before the programme**	**N° of beds**	**N° of bed nets distributed**	**% of nets coverage**	**N° nets retreated**	**% nets retreated**
Bilingue	56	17	150	153	100%	94	61.4%
Membrat	1350	14	2627	1703	64.8%	1100	64.6%
Plateau	195	0	489	475	97.1%	320	67.4%
Gare	98	0	182	123	67.6%	52	42.3%
Total	1699	31	3400	2454	72.2%	1566	63.8%

N° : number; % : proportion.

### Mosquito distribution

A total of 6959 anophelines were collected during the study, 6029 in Mbandjock and 930 in Nkoteng. The 6029 anophelines recorded in Mbandjock were collected by 1260 men-night catches (corresponding to 1050 anophelines by 201 men-nights in Bilingue, 2172 anophelines by 561 men-nights in Membrat, 2177 anophelines by 229 men-nights in Gare, and 630 anophelines by 269 men-nights in Plateau) whereas the 930 anophelines of Nkoteng were collected by 213 men-nights catches. Seven species were recorded in both Mbandjock and Nkoteng: *Anopheles coustani, An. funestus, An. gambiae sl, An. moucheti, An. ziemanni, An. nili* and *An. paludis*. *An. gambiae* was the most prevalent species representing 75.6% and 86.6% of the total anopheline caught before and after net coverage respectively in Mbandjock (Table 2). Three members of the *An. gambiae* complex were found in Mbandjock: *An. gambiae* S molecular form the predominant species (96%), *An. gambiae* M molecular form (1%) and *An. arabiensis* (3%). Molecular analysis conducted with specimens from the *An. funestus*, *An. nili* and *An. moucheti* groups using available diagnostic tools [21,22,26], confirmed the presence of *An. funestus ss, and the type forms of An. nili* and *An. moucheti* in Mbandjock and Nkoteng (Table 3).

**Table 2:** Distribution of anopheline species collected in Mbandjock and Nkoteng from January 1997 to September 1998 before and after bed net distribution

	**Mbandjock**										**Nkoteng**	
	**Before**					**After**					**Before**	**After**
**Species**	**Bilingue**	**Gare**	**Membrat**	**Plateau**	**Total (%)**	**Bilingue**	**Gare**	**Membrat**	**Plateau**	**Total (%)**	**n (%)**	**n (%)**
An. coustani	0	3	0	0	3 (0.1%)	13	52	4	9	78 (2.4%)	0 (0%)	13 (1.63%)
An. funestus	66	77	99	48	290 (10.27)	31	48	27	44	150 (4.68%)	56 (41.48%)	472 (59.37%)
An. gambiae s.l.	431	612	977	113	2133 (75.6%)	389	1114	957	315	2775 (86.6%)	61 (45.19%)	274 (34.46%)
An. moucheti	62	30	27	27	146 (5.17%)	4	9	4	1	18 (0.56%)	2 (1.48%)	7 (0.88%)
An. ziemanni	3	27	3	4	37 (1.31%)	0	0	0	0	0 (0%)	6 (4.44%)	3 (0.38%)
An. nili	31	86	56	30	203 (7.2%)	19	119	18	29	185 (5.77%)	10 (7.4%)	22 (2.77%)
An. paludis	1	0	0	10	11 (0.39%)	0	0	0	0	0 (0%)	0 (0%)	4 (0.5%)
Total	594	835	1162	232	2823 (100%)	456	1342	1010	398	3206 (100%)	135 (100%)	795 (100%)

Before: Before bed net coverage; After: After bed net coverage; n: number collected; %: percentage.

**Table 3:** **Molecular identification of members of the *Anopheles gambiae *complex, *An. funestus*, *An. nili *and *An. moucheti *groups in Mbandjock and Nkoteng**

	***An. gambiae *complex**			***An. funestus *group**	***An. nili *group**	***An. moucheti *group**
**Site**	***An. gambiae *M form**	***An. gambiae *S form**	***An. arabiensis***	***An. funestus***	***An. nili *(type form)**	***An. moucheti *(type form)**
Nkoteng	1/73 (1,4%)	70/73 (95.9%)	2/73 (2.7%)	150/150 (100%)	20/20 (100%)	20/20 (100%)
Mbandjock	1/69 (1.4%)	66/69 (95.6%)	2/69 (2.9%)	25/25 (100%)	20/20 (100%)	20/20 (100%)

### Biting behavior

**Figure 2:**
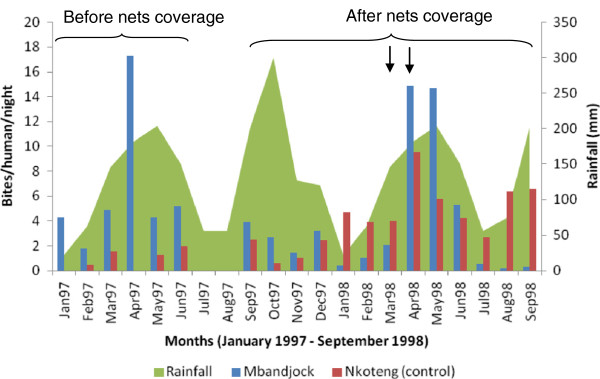
Monthly variation of mosquito biting rate in the city of Mbandjock and Nkoteng before and after cyfluthrin impregnated nets coverage (the arrows indicate the period of net retreatment).

Before bed net distribution, the human biting rate (HBR) varied from 1.79 to 17.3 bites/human/night in Mbandjock (Figure 2). The highest HBR (17.3 bites/human/night) was recorded during the month of April 1997 and was closely associated to increased mosquito frequency in the districts of Bilingue (36 bites/human/night) and Gare (34.75 bites/human/night). In the districts of Membrat and Plateau, the average HBR during the same period varied from 1.67 to 9.68 bites/human/night and 0.937 to 2.79 bites/human/night respectively.

After impregnated net coverage, the average HBR was low from September 1997 to March 1998 (< 4 bites/human/night). The highest HBR was recorded in April and May 1998 with over 14 bites/human/night during each month (Figure 2). When the data was split into districts, Bilingue with >50 bites/human/night in April 1998 and Gare with >20 bites/human/night in May 1998 scored the highest HBR. A decrease of vector HBR was recorded from June to September 1998 in all districts.

*An. gambiae*, the main species in Mbandjock was responsible for an average HBR of 6.3 before bed nets to 2.85 bites/human/night after bed net coverage. The average HBR for the other species were 0.85, 0.44 and 0.59 before bed net coverage and 0.13, 0.02 and 0.2 bites/human/night after bed net coverage for *An. funestus, An. nili* and *An. moucheti* respectively.

HBR in the city of Nkoteng was estimated to be 0 to 2 bites/human/night before bed net coverage and 0.6 to 9.5 bites/human/night after bed nets distribution (Figure 2). The HBR of species collected in the area was 1.37, 1.46, 0.049 and 0.24 before net coverage and 2.34, 1.34, 0.04, 0.12 after net coverage for *An. gambiae, An. funestus, An. nili* and *An. moucheti* respectively.

The ratio of mosquito biting indoors in Mbandjock was 55.2% and 69.5% before and after bed net coverage respectively. The difference between these percentages was significant (*χ*^2^ = 118.7; df = 1 P < 0.0001). A similar trend was recorded in the site of Nkoteng where a significant difference was recorded between the proportion of mosquitoes feeding indoors before (25.5%) and after (69.4%) bed net distribution (*χ*^2^ = 36.6; df = 1 P < 0.0001). These data suggest no repulsive effect of cyfluthrin on mosquitoes.

### Night biting cycle

**Figure 3:**
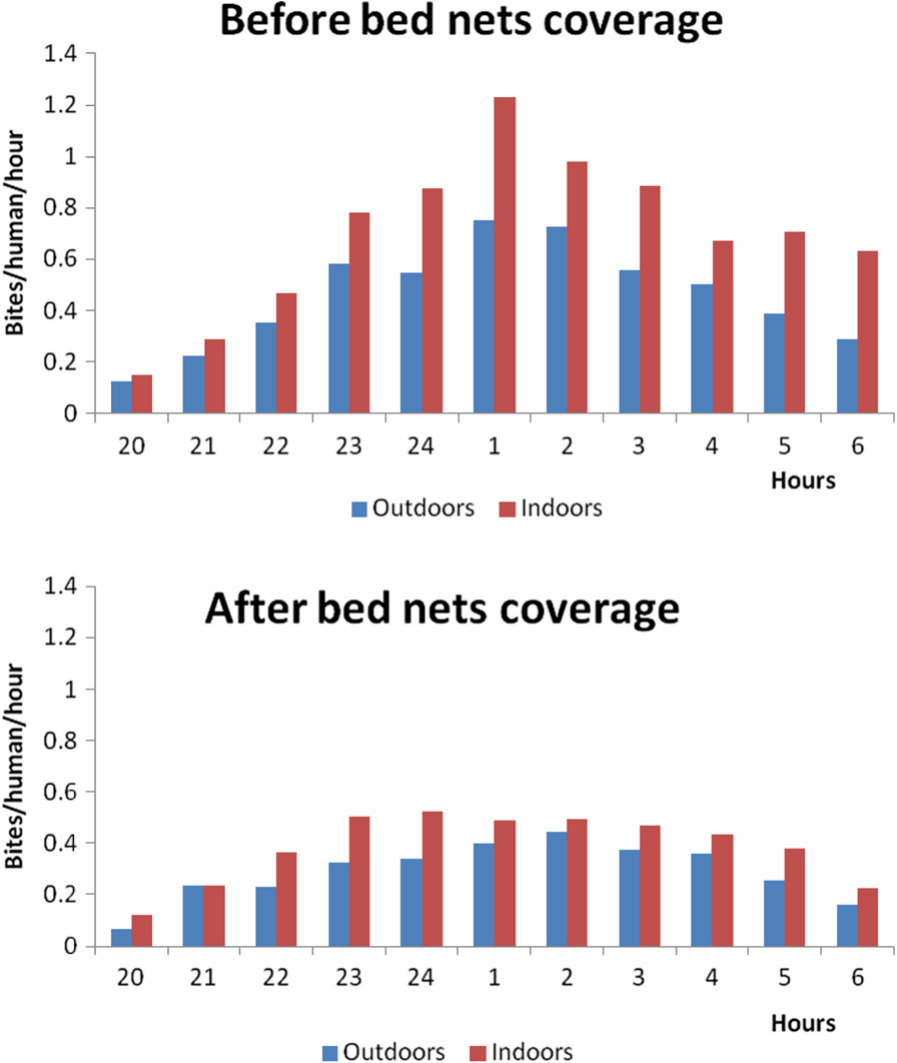
Graphs showing night biting cycle of mosquitoes in and outdoors before and after bed net distribution in Mbandjock.

No variation was recorded in the night biting cycle of mosquitoes biting in or outdoors before or after impregnated bed net distribution (Figure 3). However, a decrease in the frequency of bites was recorded after bed net coverage. The maximum number of bites was less than 0.5 bite/human/hour after net coverage while it was estimated to be over 0.8 bite/human/hour before bed net distribution.

### Parity rate

A total of 4927 female anophelines in Mbandjock corresponding to 2440 collected before bed net distribution and 2487 after bed net distribution, were dissected to determine their parity rate. The parity rate was significantly higher before (50.45%) as compared to after bed net distribution (39.89%) (*χ*^2^ = 55,07; df = 1; P < 0.0001). When the parity rate of mosquitoes in each district was compared before and after net distribution, the proportion of parous females in the majority of the districts (Gare, Membrat and Plateau), was significantly higher before as compared to after bed net distribution (P < 0.001), indicating a reduction of mosquito longevity (Table 4). In the city of Nkoteng, which was free of nets, the parity rate was not significantly different before (82.8%, 101/122) and after bed net coverage (73.8% 442/599) (*χ*^2^ = 3.94; df = 1; P = 0.05).

**Table 4:** Comparison of mosquito parity rate before and after bed net distribution in the districts of Mbandjock and Nkoteng

	**Parity rates**				
**Site**	**Before bed nets**		**After bed nets**		**Pvalue**
	**N**	**%**	**N**	**%**	
Bilingue	540	45%	413	44.07%	0.83
Gare	657	55.86%	806	42.06%	<0.0001
Membrat	1033	48.89%	917	35.99%	<0.0001
Plateau	210	55.24%	351	40.17%	<0.0007
Average Mbandjock	2440	50.45%	2487	39.89%	<0.0001
Nkoteng	122	0.83%	599	73.79%	0.05

P significant if <0.05; N: total parous and nulliparous females.

### Infection rate

Out of the 5537 mosquitoes dissected in Mbandjock, 173 were found carrying *Plasmodium* sporozoites in their salivary glands (107 of 1906 before bed net distribution and 66 of 3631 after bed net distribution). Four species were found to be infected with *Plasmodium falciparum* sporozoites: *Anopheles gambiae, An. funestus, An. moucheti* and *An. nili* (Table 5). The average infection rate in Mbandjock significantly decreased from 5.3% (107/1906) before bed net distribution to 1.8% (66/3631) after bed net distribution (*χ*^2^ = 54.09; df = 1; P < 0.0001). While in Nkoteng, the infection rate before (16%, 9/56) and after impregnated bed net distribution (8.8%, 67/762) was not significantly different (*χ*^2^ = 2.47; df = 1; P = 0.12). Because at the time the study was conducted, our laboratory lacked the necessary facility for ELISA analysis, a subsample of 50 specimens found positive after dissection including 40 samples from Mbandjock (10 from each district), exclusively *An. gambiae* and, 10 from Nkoteng consisting of 5 *An. gambiae* and 5 *An. funestus* were tested later by ELISA. The ELISA analysis confirmed all mosquitoes as positives and detected the presence of only *Plasmodium falciparum* in infected mosquitoes.

**Table 5:** Infection rate of mosquitoes collected in Mbandjock and Nkoteng before and after cyfluthrin impregnated net distribution

	**Before nets coverage**				**After nets coverage**			
	**Mbandjock**		**Nkoteng**		**Mbandjock**		**Nkoteng**	
**Species**	**Diss(Infec)**	**% (95%CI)**	**Diss(Infec)**	**% (95%CI)**	**Diss(Infec)**	**% (95%CI)**	**Diss(Infect)**	**% (95%CI)**
*An. gambiae*	1369 (82)	6% (4.8-7.4)	25 (7)	28% (11.3-57.7)	3131 (56)	1.8% (1.3-2.3)	256 (41)	16% (11.5-21.7)
*An. funestus*	177 (18)	10.2% (6–16)	5 (1)	20% (0.5-111.4)	207 (6)	2.9% (1.06-6.3)	453 (26)	5.7% (3.7-8.4)
*An. moucheti*	103 (4)	3.9% (1.06-9.9)	2 (0)	0	50 (1)	2% (0.05-111.4)	7 (0)	0
*An. nili*	140 (3)	2.1% (0.4-6.3)	5 (1)	20% (0.5-111.4)	227 (3)	1.3% (0.3-3.9)	24 (0)	0
Others	11 (0)	0	1 (0)	0	82 (0)	0	22 (0)	0
Total	1906 (107)	5.6% (4.6-6.8)	56 (9)	16.1% (7.3-30.5)	3631 (66)	1.8% (1.4-2.3)	695 (67)	9.6% (7.5-12.2)

Diss: number dissected, Infec: number found carrying *Plasmodium* sporozoites, 95% CI: 95% Confidential Interval.

### Malaria transmission

The entomological inoculation rate in Mbandjock was estimated to be 0.34 infected bites/human/night before bed net distribution and, 0.089 infected bites/human/night after bed net distribution. The use of bed net in Mbandjock reduced the risk of malaria being transmitted during a night by 74%. By making approximations on a whole year, it appeared that the annual malaria transmission in Mbandjock could be estimated to be 124.1 infected bites/human/year before cyfluthrin bed net distribution and 32.5 infected bites/human/year after cyfluthrin bed net distribution. The reduction of the EIR was highest (>85%) in the district of Bilingue, followed by Membrat (>79%). A low reduction of malaria transmission was recorded in the districts of Plateau and Gare, 69.1% and 54.54% respectively and was probably due to the low utilization of bed nets by the population in these districts (Table 6). When considering the contribution of each species to malaria transmission before bed net distribution, it appeared that *An. gambiae* was responsible for 77% of malaria transmission followed by *An. funestus, An. moucheti* and *An. nili* responsible for 16.7%, 3.5% and 2.6% of malaria transmission respectively. After bed net coverage, *An. gambiae* was again responsible for the highest share of malaria transmission (88%). *An. funestus, An. moucheti* and *An. nili* were responsible for 7%, 0.7% and 4.5% of malaria transmission.

**Table 6:** Estimates of daily entomological inoculation rate (EIR)recorded in Nkoteng and districts of Mbandjock before and after bed net coverage

	**Daily EIR**		
**Sites**	**Before bed nets**	**After bed nets**	**Reduction of EIR**
Bilingue	0.464	0.066	85.86%
Gare	0.368	0.167	54.54%
Membrat	0.372	0.075	79.77%
Plateau	0.155	0.048	69.1%
Average Mbandjock	0.339	0.089	73.81%
Nkoteng	0.18	0.37	−105%

**Figure 4:**
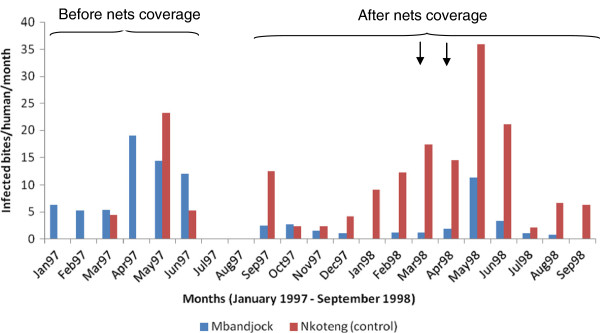
Monthly variation of the entomological inoculation rate in the cities of Mbandjock and Nkoteng before and after cyfluthrin bed net coverage (the arrows indicate the period of net retreatment).

The entomological inoculation rate in Nkoteng was 0.18 and 0.37 infected bites/human/night before and after bed net distribution respectively (corresponding to 65.8 infected bites/human/year before bed net coverage and 146.6 infected bites/human/year after nets coverage). Malaria transmission in Nkoteng was exclusively due to *An. funestus* and *An. gambiae,* being responsible for 41.6% and 58.4% of malaria transmission before bed net coverage and 38.6% and 61.4% after bed net coverage respectively. Figure 4 gives a general pattern of the monthly variation of malaria transmission in the cities of Mbandjock and Nkoteng.

## Discussion

A decline of malaria morbidity and mortality has been reported during the past decade in several sub-Saharan countries [27,28], whereas, the disease still has a devastating impact on public health and welfare in Cameroon [2,29,30]. The situation requires a thorough assessment of factors affecting directly or indirectly control operations in order to find lasting solutions. Revisiting past control operations could provide useful guidelines for current or future initiatives. In the locality of Mbandjock, a decrease of malaria transmission with high variation between districts was detected after the distribution of cyfluthrin impregnated nets. The district of Bilingue with a 100% bed net coverage rate, scored the highest decrease of malaria transmission. No reduction of malaria transmission was recorded in Nkoteng, which was bed net free. Malaria transmission estimates recorded in Nkoteng were slightly lower than data reported by Cohuet *et al.*[31]. In addition to seasonal fluctuations, this difference might probably result from the fact that in their study, Cohuet *et al.*[31] included samples from the city periphery while our study was limited to the city centre. Several factors including human activities, seasonal fluctuations, the practice of agriculture, irrigation or environmental modifications can influence vector distribution and malaria transmission patterns making it possible that transmission patterns could vary significantly within the same city or from one city to the other [32-36]. However, the reduction of malaria transmission in Mbandjock following bed net distribution was consistent with the known impact of pyrethroid impregnated nets on malaria transmission [37-40]. The potential epidemiological advantages of using pyrethroid treated nets in public health include reduction of childhood and overall mortality and reduction of malaria morbidity [17]. Although a low level of pre-existing net usage was registered before the programme, a high acceptability of bed net use by the population was recorded. A decrease of several parameters was detected respectively, HBR, parity rate, infection rate and entomological inoculation rate amongst others. The decrease of HBR was consistent during the seven months following bed net coverage before increasing. This highlights the need for regular retreatment of non-long lasting insecticidal nets every six months. Although up to 64% of nets were retreated in the course of the study, very low retreatment rates have usually been reported in communities [16]. Conscious of these limits and the low bed net ownership, the Cameroon government, opted for the free distribution of long lasting nets to the population [15]. Beyond the significant impact awaited from this programme, it is questionable if this initiative is capable of breaking down the burden of malaria across the country. However, the distribution of LLINs will increase bed net ownership and avoid retreatment but it will need to be sustainable. Just after cyfluthrin impregnated net distribution in Mbandjock, a high utilization rate was recorded and this rate decreased subsequently following the non replacement of damaged nets or nets distributed to relatives residing out of Mbandjock. Moreover, with the decrease of mosquito burden, many people were not using nets regularly. Several behaviors hindering the use of bed nets have been recorded across the country and include the use of bed nets for fishing or the habit of sleeping outdoors without any protection during the hot periods [41]. These cases highlight the need for constant sensitization campaigns to keep people constantly vigilant about the obligation to adopt good attitudes. A decrease of the parity rate and consequently of the mosquito life span by cyfluthrin, was also scored as a positive outcome of the Mbandjock bed net programme. This data closely matches results of the bioefficacy analysis of cyfluthrin impregnated nets which reported a mortality rate on susceptible mosquitoes ranging from 60 to 100% during the first four months after bed net impregnation and a mortality rate ranging from 50 to 90% during the first 5 months after reimpregnation [25]. Although LLINs keep their efficacy even after several washings [37,38,42], a low insecticidal activity and personal protection were demonstrated in West Africa with several varieties of these nets [43]. In addition, recent field evaluation studies reported limited efficacy of LLINs against pyrethroid resistant mosquitoes [44]. These findings highlight the necessity to combine the use of LLINs with environmental management strategies in order to prevent the expansion of vector resistance to insecticides [45].

In contrast to small or middle scale projects such as the one of Mbandjock, which are limited by time and involve limited collaborations, the challenge is quite high for nationwide coverage programs. This includes developing a national LLINs strategy based on at least 4 key points: the empowerment of communities for sustainable control, assessment of the capacity of the commercial sector for regular supply of LLINs, creating an enabling environment for LLINs and establishing a partnership between public and private sectors [17]. Although Cameroon has an ITN strategic plan, the contribution and the potential of the private sector in the commercialization of nets is still underestimated while this sector could be of great support for the regular supply of nets to communities. Since the average life span of a net is up to 5 years, there is a need to look for sources for a regular supply.

In this framework, elaborating a public-private joint plan for the sustainability of universal net coverage is crucial to breakdown the malaria burden, in order to reach the millennium development goals [46]. Although some private NGOs such as ACMS (“*Association Camerounaise pour le Marketing Social”*) supported by the German cooperation contributed substantially to bed net distribution in some parts of the country [47], there is much left to be done particularly for some rural communities or towns situated some distance away from the capital city. The enrolment of communities in the supply of LLINs or the fight against malaria vectors could appear as a practical alternative as it is the case elsewhere [48,49]. The empowerment of communities in the fight against malaria vectors is fundamental. This includes encouraging social or traditional practices promoting LLINs, encouraging local production of nets, promoting sensitization campaigns against malaria in schools and in communities, supporting initiatives in favor of hygiene and sanitation in communities such as the competition of the cleanest town currently underway across the country (http://cameroun-online.com/actualite,actu-8431.html). At the difference of small scale programs which are limited by time, nationwide programs have to bring together all initiatives in order to guarantee a sustainable control of malaria.

## Conclusion

Notwithstanding the fact that it is almost 15 years since the Mbandjock treated bed net programme was undertaken and, many important improvements have been made since then in the treatment or distribution of nets, the main conclusions of this study are still relevant nowadays. Besides, the study provides information on past control activities in Cameroon, which has been generally lacking. The Mbandjock bed net project permitted the assessment of the effectiveness of cyfluthrin impregnated nets against malaria vectors. However, no provision for sustainability was made. These limits have to be taken into consideration by nationwide programmes to ensure proper efficiency and sustainability of national control strategies.

## Competing interests

The authors declare that they have no competing interests.

## Authors’ contributions

Conceived and designed the study protocol: BB. Participated in field and laboratory analyses, CAN, MD, JE, BB. Interpreted, analyzed data and wrote the paper: CAN. All the authors read and approved the final version of the manuscript.
